# Dynamic miRNA–host gene co-expression and functional regulation in response to salinity fluctuations during biological invasions

**DOI:** 10.1007/s42995-026-00361-w

**Published:** 2026-03-05

**Authors:** Weijie Yan, Ruiying Fu, Xuena Huang, Noa Shenkar, Aibin Zhan

**Affiliations:** 1 Research Center for Eco-Environmental Sciences, Chinese Academy of Sciences, Beijing, 100085 China; 2 Chinese Academy of Sciences, University of Chinese Academy of Sciences, Beijing, 100049 China; 3 School of Zoology, George S. Wise Faculty of Life Sciences, Tel-Aviv University, Tel-Aviv, 6997801 Israel; 4 The Steinhardt Museum of Natural History, Israel National Center for Biodiversity Studies, Tel Aviv University, Tel-Aviv, 6997801 Israel

**Keywords:** Intragenic miRNA, Biological invasion, Co-expression, Environmental challenge

## Abstract

The significantly negative impact of marine invasive species underscores the need to understand the dynamics of invasion success. MicroRNAs (miRNAs) play a crucial role in regulating gene expression in response to stresses during invasions. Using the invasive tunicate *Ciona robusta* as a model, here we aim to study intragenic miRNA–host gene co-expression and functional regulation in response to recurrent salinity challenges. Despite genomic nestedness, only 9% of miRNA–host gene pairs showed significant co-expression (*p* < 0.05, correlation coefficient > 0). Recurring stresses dynamically altered the co-expression, revealing distinct miRNA–host gene expression at different stress times and stages. These differentially expressed miRNAs (*p*_*adj*_ < 0.05, |log_2_foldchange|> 1) regulated biological processes, including free amino acid metabolism, water channel function, and ion transport to maintain osmotic homeostasis. These functional regulations were specific to time and stage, targeting the same type of osmolytes through varied pathways. Our findings highlight the diverse regulatory roles of miRNAs in enabling rapid responses to environmental stresses during invasions, providing new insights into miRNA-driven phenotypic plasticity under changing conditions.

**Supplementary Information:**

The online version contains supplementary material available at 10.1007/s42995-026-00361-w.

## Introduction

Biological invasions pose significant challenges worldwide, disrupting ecosystems, threatening biodiversity, and causing economic losses and health risks (Diagne et al. 2021; Simberloff et al. 2013). Despite extensive management efforts, human activities and global changes have accelerated invasion rates, exacerbating their impacts at all scales (Chen et al. 2024; Crystal-Ornelas et al. 2021; Diagne et al. 2021; Li et al. 2024). Economic costs from biological invasions rose 702% from 1980–1999 to 2000–2019, surpassing damages from natural hazards such as storms (Turbelin et al. 2023). Aquatic systems, particularly freshwater and marine, are among the most affected, with costs reaching at least US$23 billion in 2020 (Cuthbert et al. 2021). These impacts underscore the need for innovative management strategies and a deeper understanding of invasion dynamics (Chen et al. 2024; Liu et al. 2024; Zhan 2025).

During the invasions process, invasive species often encounter extreme environments, and recurrent and/or sudden environmental changes pose significant threats to their survival (Bereza et al. 2022; Briski et al. 2013; Huang et al. 2023a). To succeed during the invasion process, invaders must activate their own multi-level adjustments for phenotypic plasticity, such as rapid physiological and biochemical regulations to improve their fitness (Huang et al. 2021; Li et al. 2024; Richards et al. 2006). From a molecular perspective, one crucial common component responsible for phenotypic plasticity is gene expression as effective regulatory networks for gene expression enable species to swiftly alter both the composition and concentration of macromolecules such as proteins to adjust their biological processes according to ever-changing environments (Bossdorf et al. 2008; Jonsson and Jonsson 2019; Hill et al. 2021; Huang et al. 2023a). Among intricate regulatory networks for gene expression, microRNAs (miRNAs) are recognized as the sculptor of transcriptomes as they play essential roles in nearly all biological processes and post-transcriptionally regulate more than 1/3 protein-coding genes (Friedman et al. 2009; Solís et al. 2022). Moreover, recent studies have further highlighted the diversified multifunctional nature of miRNAs. By binding to diverse regions of target genes, including the 3' untranslated region (UTR), 5' UTR, coding region, promoter, and enhancer, miRNAs can both up- and down-regulate the expression of target genes (Pu et al. 2019; Wu et al. 2013; Yan et al. 2025a). Such versatile regulations can fine-tune the timing and magnitude of the expression of target genes, adjusting protein abundance to meet the specific demands of biological processes within harsh environments (Rani et al. 2022; Yan et al. 0.2025b). Given the functional roles of miRNAs, the process of miRNA biogenesis, which determines their abundance in cells, is one crucial process of miRNA-mediated gene expression regulation.

**Fig. 1:**
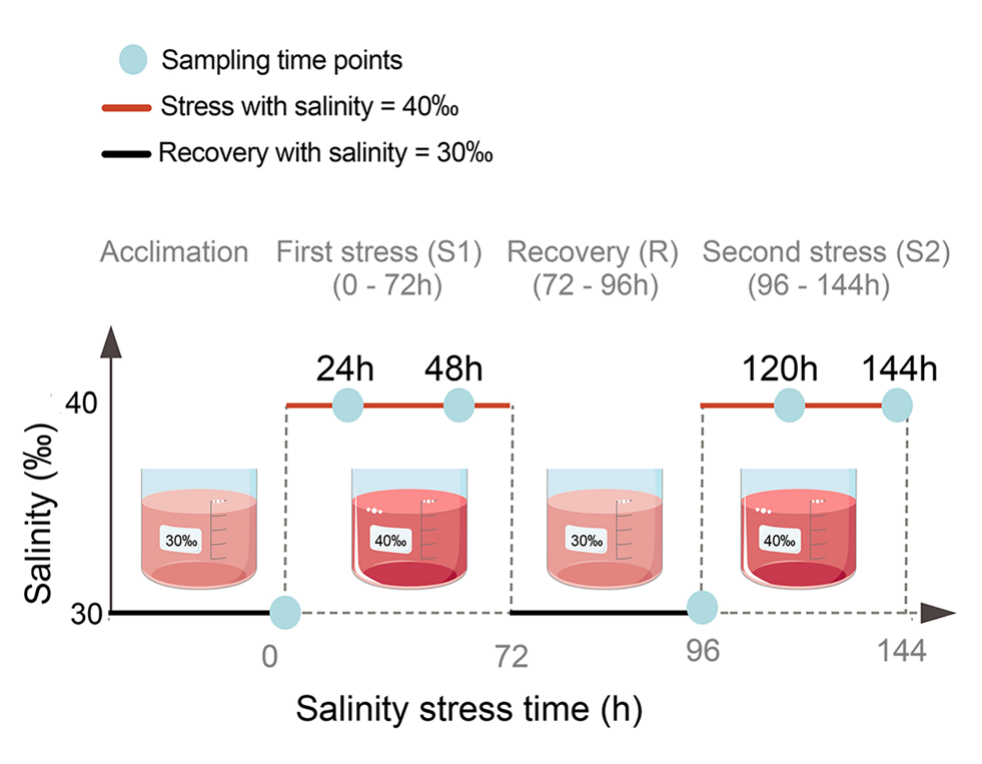
Illustration of the experimental design. After acclimation, healthy *Ciona robusta* individuals were randomly selected for the recurrent hyperosmotic stress. *C. robusta* individuals were treated with hyperosmotic stress (40‰) for 72 h (the first stress, S1 stage) and sampled at 0, 24, and 48 h; transferred into the ambient condition (30‰) for 24 h (Recovery, R stage) and sampled at 96 h; and finally subjected to another round of hyperosmotic stress (40‰) for 72 h (the second stress, S2 stage) and sampled at 120 and 144 h

Among the factors that influence miRNA biogenesis, the genomic location of miRNAs largely determines how primary miRNAs (pri-miRNAs) are transcriptionally regulated (Gao et al. 2012; Rani et al. 2022). According to their genomic position, miRNAs can be classified into intragenic and intergenic categories—intragenic miRNAs are embedded within introns (intronic miRNAs), exons (exonic miRNAs), intron–exon junctions (junction miRNAs) of host genes on the same strand, as well as the antisense strand (antisense miRNAs), while intergenic miRNAs are situated between genes (Liu et al. 2019a). Compared to intragenic miRNAs, the transcription of intergenic miRNAs is more straightforward and less complex. Most intergenic miRNAs use their own promoters to independently generate pri-miRNAs, while some have been observed to be co-transcribed with neighboring genes through several additional mechanisms, such as readthrough and divergent transcription (Caldas et al. 2024; Georgakilas et al. 2014). These co-transcribed miRNAs are sometimes classified into the intragenic category, mainly because they potentially share the same promoters with their neighboring genes (Liu et al. 2019a). However, accumulating evidence has clearly illustrated complex dynamics in the transcriptional regulation of intragenic miRNAs by their host genes (Rani et al. 2022; Schanen et al. 2011). Early studies support the co-expression model for intragenic miRNAs and their host genes, demonstrating that the common primary transcripts, transcribed from the transcriptional start sites (TSSs) of their host genes, are processed into both mRNA and miRNAs through microprocessor cleavage and splicing (Baskerville et al. 2005; Kim et al. 2007). However, intensive studies have shown that the co-expression model is not always as expected, and the transcription and associated regulation vary depending on the subtypes of intragenic miRNAs. For intronic miRNAs, the presence of independent miRNA promoters, interactions between microprocessor cleavage and splicing, and even variations in evolutionary conservation levels of miRNAs have been observed to often disrupt the co-expression pattern (Agranat-Tamir et al. 2014; He et al. 2012; Schanen et al. 2011). Because exonic miRNAs are embedded within exons, pri-miRNA cleavage by Drosha disrupts the splicing of host mRNAs, resulting in distinct transcriptional regulation compared to intronic miRNAs (Sundaram et al. 2013). Depending on whether the splicing machinery recognizes the internal exon, the biogenesis of junction miRNAs is the outcome of competition between the spliceosome and microprocessor, making the biogenesis the most complicated (Melamed et al. 2013). These diversified regulatory dynamics allow intragenic miRNAs to function both in conjunction with and independently of their host genes, enhancing their versatility and flexibility in diversely regulating the expression of their target genes.

**Fig. 2:**
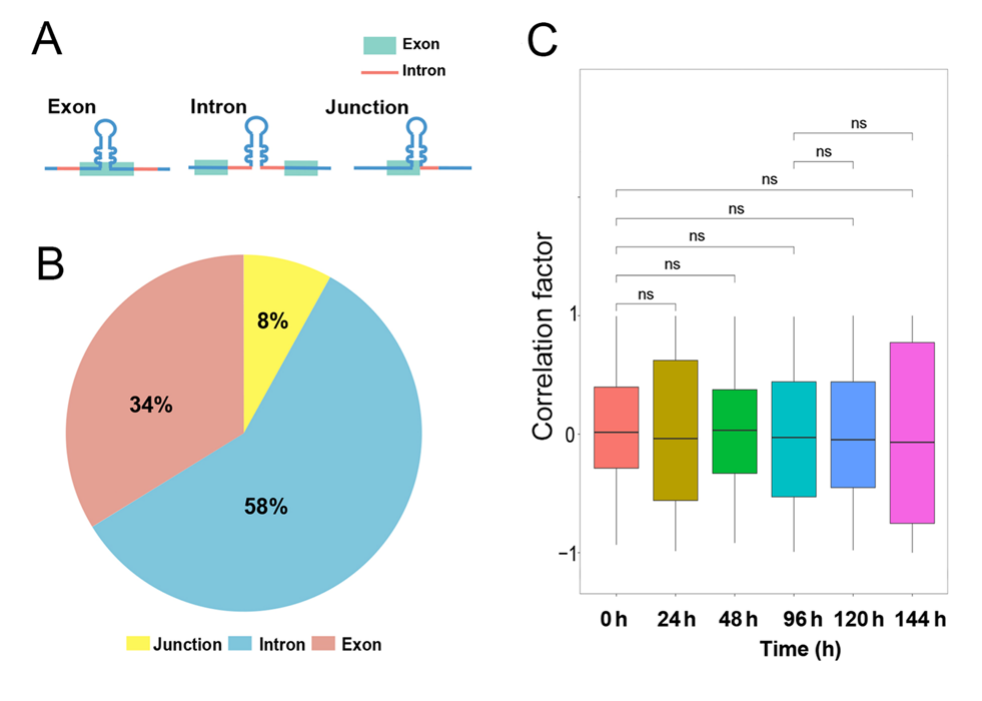
**A** Genomic distribution of intragenic miRNA genes in *Ciona robusta*. The three types of genomic location (exon, intron, and junction) were labeled in distinct colors. **B** Visualization of three types of genomic location of intragenic miRNA genes. **C** Global correlation coefficients of co-expressed miRNAs and corresponding host genes at different sampling points (0, 24, 48, 96, 120, and 144 h). Differences between 0 h and other time points (96 and 120 h, and 96 and 144 h) were estimated using the Wilcoxon test (*p* < 0.05)

To date, almost all studies on the interplay between miRNAs and their host genes have been conducted in model species or humans, with this interplay being implicated in the occurrence of diseases such as cancer (Liu et al. 2019a). However, the findings from these studies indicate that intragenic miRNAs regulated by their host genes play a role in responding to environmental challenges within ecological and evolutionary contexts, such as biological invasions. Unlike studies in model organisms or humans, investigating the interplay between miRNAs and their host genes, particularly their functional roles in gene expression regulation, faces significant challenges in ecological and evolutionary studies, such as the pulse of extreme environmental changes, diverse interactions between the complexity of environmental contexts and interplay of miRNA–host genes. Meanwhile, both knowledge and methodology on this topic is notably lacking, adding an additional layer of technical difficulty in ecological and evolutionary studies. Thus, selecting a promising system is crucial to deeply understand causes and consequences of miRNA–host genes interactions in response to environmental changes during biological invasions.

*Ciona robusta*, formerly known as sp. A in the *C. intestinalis* species complex (Brunetti et al. 2015), is a highly invasive ascidian presumed native to the Northwest Pacific (Caputi et al. 2007; Zhan et al. 2015). *C. robusta* has successfully invaded global coasts, including the extreme environment of the Red Sea, causing substantial economic loss and ecological issues in these habitats (Chen et al. 2018; Shenkar et al. 2018; Zhan et al. 2015). Owing to its biological features, such as high fecundity, significant invasiveness, high environmental tolerance, strong adaptive capacity, and a well-sequenced small genome, *C. robusta,* along with its congener *C. intestinalis*, has become a model system for studying the mechanisms underlying invasion success (Zhan et al. 2015). Using this model system, previous studies have confirmed functional responses of gene expression to environmental challenges, such as the regulation of osmotic pathways for various substances, including metal ions and free amino acids, in response to salinity stresses (Bereza et al. 2022; Huang et al. 2023a, b). Stressed populations can develop a “stress memory”, enabling more accurate and effective responses when they subsequently experience the same or similar stresses (Li et al. 2020; 2024). Further investigations into the mechanisms underlying functional gene expression changes revealed dynamic transcriptional reprogramming and length shifts of responsive isoforms directed by multiple processes, such as epigenetic and post-transcriptional regulations (Bereza et al. 2022; Fu et al. 2021a; Huang et al. 2023b). For examples, osmotic challenges rapidly triggered epigenetic responses, such as genome-wide DNA methylation changes within 1 h, which further regulated the expression of genes involved in proline biosynthesis, lipid metabolism, and taurine and Ca^2+^ transport (Fu et al. 2021a; Huang et al. 2017). Post-transcriptional regulations, such as alternative splicing and alternative polyadenylation, up-regulated isoforms with additional transmembrane regions in genes, such as *SLC2a5* and *Cyb5r3*, to enhance transport activities (Huang et al. 2021, 2023b). However, integrated analysis of multi-omics datasets showed that only a small proportion of genes were regulated by these processes, indicating that the regulatory networks are more complex than initially expected (Huang et al. 2023a). Thus, incorporating additional regulatory processes, such as those mediated by miRNAs, is essential for deeply understanding the complex gene expression networks that respond to environmental challenges during biological invasions.

Using the model invasive ascidian *C. robusta*, we simulated recurring hyper-salinity stress encountered during the invasion process and integrated miRNAomic and transcriptomic data to analyze the co-expression patterns of intragenic miRNAs and their host genes. Given the diverse regulatory relationships between intragenic miRNAs and their host genes, as well as miRNAs’ functional roles in target gene expression, the present study aimed to test whether recurring environmental stresses significantly affect the co-expression between intragenic miRNAs and their host genes and whether any altered co-expression leads to downstream functional changes in the expression of miRNA target genes in response to environmental challenges. The results of this study are expected to provide deeper insights into the complex miRNA-modulated regulatory networks involved in gene expression, a key driver of phenotypic plasticity in response to environmental challenges during biological invasions and other extreme events such as those derived from global change.

## Materials and methods

### Experimental design, sample, and sequencing

Adult *C. robusta* was collected from the artificially established substrates in Longwangtang, Dalian, Liaoning Province, China (38°49′19’’N, 121°2′28’’E; water temperature: 22 °C; salinity: 30‰). Healthy individuals were selected for subsequent experiments after a 3-day acclimation period at 22 °C in the laboratory, where they were nourished with *Spirulina* sp. and *Chlorella* sp. Salinity is a major environmental factor determining the geographical distribution of marine organisms and affecting biological processes, such as reproduction and development; this study focused on salinity stress. The ascidians were subjected to recurrent hyperosmotic stress to simulate the environmental challenges that they have encountered during invasions (Fig. 1). The control and recovery salinity levels were maintained at 30‰, consistent with conditions at the sampling site, whereas the hyperosmotic salinity was set at 40‰, reflecting the highest salinity of the Red Sea within the invasive range of *C. robusta* (Chen et al. 2018). The recurrent salinity treatment was established as follows based on our previous studies (Fu et al. 2021b; Huang et al. 2017, 2023b; Li et al. 2020): after acclimation, six individuals were collected as the control group (C), while all remaining ascidians were exposed to the high salinity for 72 h (S1 stage), followed by a return to 30‰ for 24 h (R stage), and then re-exposed to the high salinity for an additional 72 h (S2 stage). According to the findings from our previous studies on gene expression patterns (Huang et al. 2017, 2018; Li et al. 2020), sampling time points were selected based on significant changes in gene expression: 0 h at the end of acclimation (Control), 24 and 48 h during the S1 stage, 96 h during the R stage, and 120 and 144 h during the S2 stage, with six individuals sampled at each time point. Total RNA was extracted from somatic muscle tissue using Trizol reagent (Ambion, Massachusetts, USA) according to the manufacturer’s instructions for RNA-seq and miRNA-seq analyses. Both the miRNAomic and transcriptomic data have been deposited in the National Center for Biotechnology Information (NCBI) Sequence Read Archive (SRA) under accession number PRJNA775866. For transcriptome analysis, quality control was performed with FastQC v0.11.9 and low-quality reads and adapters in the raw data were trimmed using Trimmomatic v0.39. Subsequently, the expression levels of mRNAs were quantified and normalized from raw read counts to transcripts per million (TPM) (Bolger et al. 2014). The methods for analyzing transcriptome data to determine gene expression levels were detailed in our previous study (Fu et al. 2021a).

### miRNA identification and expression analysis

Due to the complex and diverse co-expression patterns among different types of miRNAs and their host genes, coupled with the limited research background and evidence in ecological and evolutionary studies, we focused on three subtypes of intragenic miRNAs, including intronic, exonic, and junction miRNAs (Fig. 2A), which exhibit tighter co-expression with their host genes. Low-quality tags containing more than one base with a *Q*-value ≤ 20 or including unknown nucleotides (N) were removed. The remaining clean tags from the miRNA-seq data were then mapped to the *C. robusta* genome (HT version, http://ghost.zool.kyoto-u.ac.jp/default_ht.html) using Bowtie2 v2.5.1. Subsequently, miRNAs were identified and quantified using the default parameters of miRDeep2, and low-frequency miRNAs, defined as those expressed in less than 50% of the samples, were excluded from further analyses (Friedländer et al. 2012; Langmead et al. 2012; Martin 2011). The identified miRNAs were classified as intronic, exonic, and junctional according to their genomic locations (Fig. 2B). The distribution of these miRNA types was visualized as a pie chart using the OmicShare platform (www.omicshare.com/tools). Differentially expressed miRNAs (DEmiRNAs) were identified between the control stage (0 h) and other sampling points (24, 48, 96, 120, and 144 h) and between the R (96 h) and S2 stages (120 and 144 h), using the “DESeq2” package with a significance threshold of *p*_*adj*_ < 0.05 and |log_2_foldchange|> 1 (Love et al. 2014).

### Co-expressions between miRNAs and host genes

As indicated in studies, the co-expression of miRNAs and host genes is evidenced by a significant correlation between the expression of miRNAs and their corresponding host genes, which can show both positive and negative relationships (He et al. 2012; Sundaram et al. 2013). Pearson correlation coefficients were calculated to assess the transcriptional relationships between intragenic miRNAs and their corresponding host genes at each sampling point using the “psych” package in R (Revelle et al. 2015). As in many studies, a correlation threshold of > 0 was set for correlations with *p* < 0.05 (He et al. 2012; Kristensen et al. 2014; Wang et al. 2012). Significant positive correlations at any sampling point were considered co-expression relationships. The variation in global correlation coefficients and co-expressed miRNA–host gene pairs across different sampling points was analyzed using the Wilcoxon test, and comparisons were made between the control group and other sampling points, between 96 h and 120 h, as well as between 96 h and 144 h. Additionally, the correlation coefficients of all intragenic and differentially expressed miRNA–host gene pairs at various sampling points were visualized using the “ggplot2” package in R (Wickham 2011).

### Functional analysis of key miRNAs

To evaluate the function of DEmiRNAs co-expressed with their corresponding host genes, the targets of DEmiRNAs at different sampling points were predicted using the independent algorithms RNAhybrid and miRanda (http://www.microrna.org/). The overlapping targets identified by both algorithms were used for further analysis. The functions of these targets were examined in two ways. First, Gene Ontology (GO) enrichment analysis was performed using OmicShare tools (https://www.omicshare.com/tools), with a significance threshold of *p* < 0.05. Second, the targets of co-expressed DEmiRNAs related to the three canonical strategies of cellular osmotic homeostasis, including ion transport, free amino acid (FAA) metabolism and biosynthesis, and water channel regulation, were retrieved from the *C. robusta* genome. These genes were selected based on research in aquatic animals (Cao et al. 2019; Liu et al. 2019b; Lou et al. 2019; Pu et al. 2017; Towle et al. 2011; Verri et al. 2012).

## Results

### Genomic distribution of miRNA genes and global co-expression with host genes

Overall, 889 miRNAs were identified in the genome of *C. robusta*. Of these, 490 miRNA genes were found in intragenic regions, with 58% located in introns, 34% in exons, and 8% at exon–intron junctions (Fig. 2B). Among these 490 miRNA–host gene pairs, only 44 (9%) showed significant positive correlations in expression between miRNAs and their host genes (i.e., co-expression; Supplementary Table S1). After comparing the correlation coefficients of miRNA–host genes across different sampling points during the repeated stress treatment, no significant differences were observed in global correlation coefficient levels between the control (0 h) and S1 (24 and 48 h), R (96 h), or S2 (120 and 144 h) stages. Similarly, no significant changes in global correlation coefficients were detected between the R (96 h) and S2 (120 and 144 h) stages (Fig. 2C).

### Stress time-dependent co-expression patterns

**Fig. 3:**
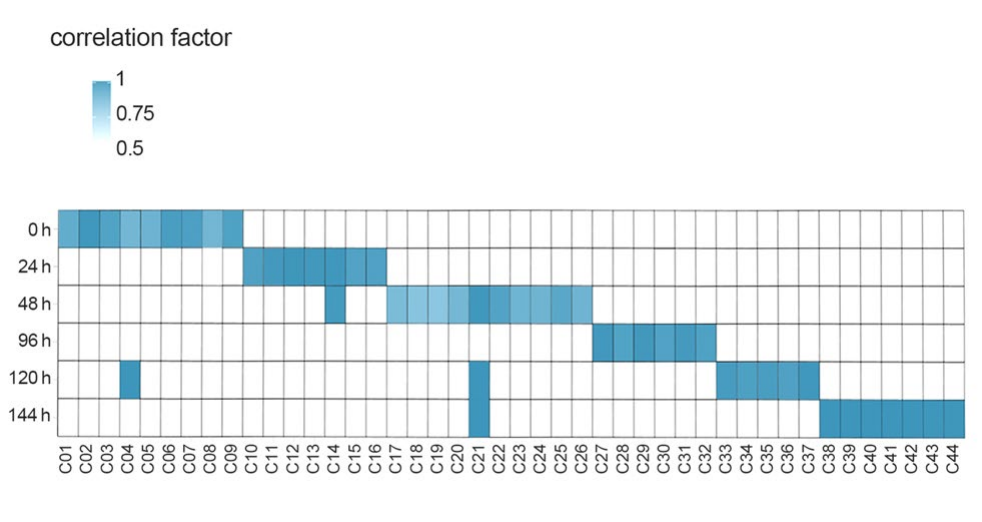
Dynamic co-expression pattern of 44 co-expressed miRNA gene-host gene pairs. The colored squares represent the co-expression time, and the color depth of the square represents the magnitude of the correlation coefficient

Interestingly, the 44 co-expressed miRNA–host gene pairs exhibited four distinct stress time-dependent co-expression patterns (Fig. 3). The first pattern involved co-expression only under non-stressed conditions, with seven miRNA–host gene pairs (C01–03 and C05–08; Fig. 3) co-expressed exclusively at the C stage; the miRNAs were transcribed independently when *C. robusta* encountered salinity challenges. The second pattern showed co-expression exclusively at the R stage (C27–32; Fig. 3). The third pattern revealed co-expression under hyperosmotic conditions, with 16 miRNA–host gene pairs co-expressed only at the S1 stage, 12 pairs only at the S2 stage, and one (C21; Fig. 3) at the S1 and S2 stages. Additionally, one miRNA–host gene pair (C04; Fig. 3) demonstrated co-expression at non-stressed and hyper-salinity (S2) stages. These patterns emphasize that recurrent salinity stresses influenced the co-expression dynamics of miRNAs and their corresponding host genes.

### Functions regulated by co-expressed DEmiRNAs

**Fig. 4:**
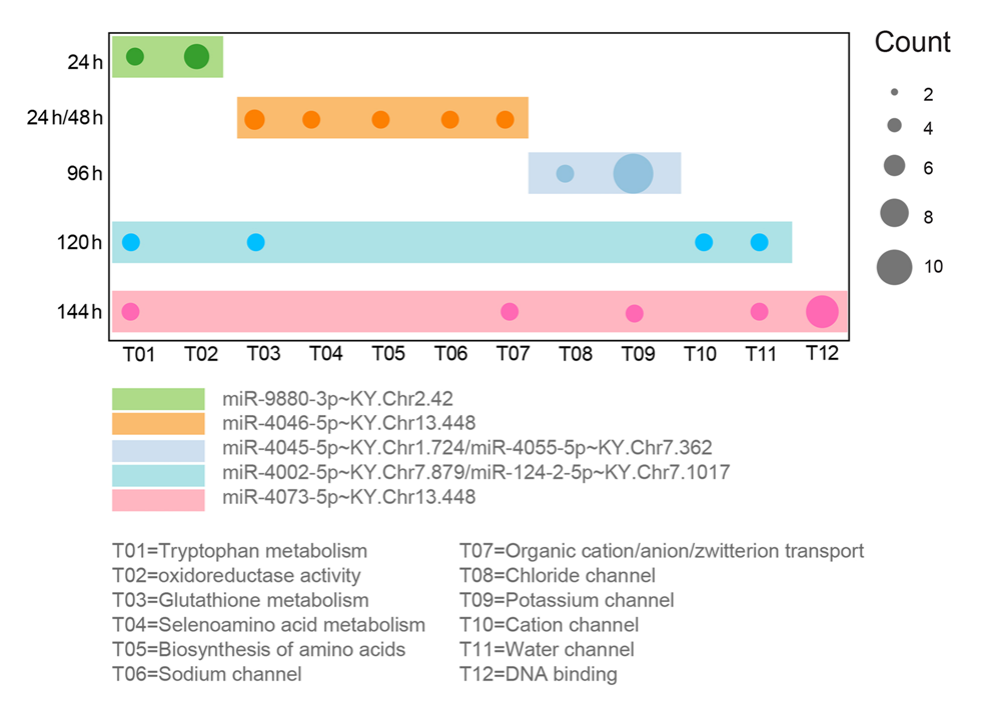
Bubble chart of 12 functions regulated by co-expressed differentially expressed miRNAs (DEmiRNAs) at different sampling points. The node size of bubbles represents the target numbers involved in terms, and DEmiRNAs co-expressed at diverse sampling points were labeled in different colors

Of the 44 miRNAs co-expressed with their corresponding host genes, seven were DEmiRNAs under osmotic stress, and stress time-dependent expression patterns were observed (Fig. 4). For example, miR-9880-3p exhibited differential expression at 24 h, and miR-4046-3p responded at both 24 and 48 h during the first round of salinity stress. Similarly, miR-4055-5p and miR-4045-5p were differentially expressed at 96 h during recovery between the two rounds of stress. Additionally, miR-4002-5p and miR-124–2-5p showed differential expressions at 120 h, whereas miR-4073-5p responded at 144 h during the second round of hyperosmotic stress (Fig. 4).

Furthermore, GO analysis and canonical strategy annotations revealed that DEmiRNAs co-expressed at the S1 and S2 stages shared some common functions, including tryptophan/glutathione metabolism and organic cation/anion/zwitterion transport. Although miR-9880-3p (24 h) and miR-124–2-5p (120 h) were involved in tryptophan metabolism, they regulated different targets: cytochrome P450 family 2 subfamily J member 2_2 (KY. Chr9.138) and cytochrome P450 family 2 subfamily J member 2_1 (KY. Chr8.177), respectively. Similarly, in the glutathione metabolism process, miR-4046-5p (24 h/48 h) regulated glutathione peroxidase 6 (KY. Chr1.1310) and glutathione S-transferase omega 2 (KY. Chr12.322), and miR-124-2-5p (120 h) regulated glutathione S-transferase theta 2B (KY. Chr10.208). These findings indicate that different DEmiRNAs can regulate distinct targets to participate in the same biological functions.

Moreover, DEmiRNAs co-expressed at the S2 and R stages regulated potassium channels: miR-4055-5p (96 h) regulated nine potassium channel-related targets, whereas miR-4073-5p (144 h) regulated potassium voltage-gated channel modifier subfamily S member 2 (KY. Chr2.1147) (Table 1). The functions of targets regulated by DEmiRNAs co-expressed at the S1 stage were associated with selenoamino acid metabolism, amino acid biosynthesis, and sodium channel regulation. In contrast, DEmiRNAs co-expressed at the R stage regulated chloride channel functions, whereas those co-expressed at the S2 stage were associated with cation channel, water channel, and DNA binding functions (Fig. 4). The functions of target genes regulated by DEmiRNAs were diverse and distinct across different sampling points during the treatment stages, with different targets being regulated to maintain the same functions at various time points.

**Table 1:** Host genes and targets of DEmiRNAs

	miRNA	Host gene	Location	Time of co-expression	Targets and function
1	cin-miR-9880-3p	KY. Chr2.42	exon	24 h	Oxidoreductase activity (KY. Chr11.206, KY. Chr8.705, KY. Chr8.706, KY. Chr8.712), Tryptophan metabolism (KY. Chr9.138)
2	cin-miR-4046-5p	KY. Chr13.448	intron	24 h/48 h	Glutathione metabolism (KY. Chr1.1310, KY. Chr12.322), sodium voltage-gated channel (KY. Chr11.378), biosynthesis of amino acids (KY. Chr5.53), selenoamino acid metabolism (KY. Chr3.1644), organic cation/anion/zwitterion (KY. Chr1.2519)
3	cin-miR-4045-5p	KY. Chr1.724	intron	96 h	Potassium channel (KY. Chr1.2098)
4	cin-miR-4055-5p	KY. Chr7.362	intron	96 h	Chloride channel (KY. Chr3.51), potassium channel (KY. Chr10.927, KY. UAContig13.17, KY. Chr47.3, KY. Chr49.3, KY. UAContig51.14, KY. UAContig51.22, KY. UAContig53.12, KY. UAContig6.54, KY. UAContig7.18)
5	cin-miR-4002-5p	KY. Chr7.879	intron	120 h	Cation channel (KY. Chr2.377)
6	cin-miR-124–2-5p	KY. Chr7.1017	exon	120 h	Glutathione metabolism (KY. Chr10.208), water channel (KY. Chr12.415), tryptophan metabolism (KY. Chr8.177)
7	cin-miR-4073-5p	KY. Chr13.448	intron	144 h	RNA polymerase II core promoter proximal region sequence-specific DNA binding (KY. Chr1.2177, KY. Chr11.469, KY. Chr12.818, KY. Chr3.226, KY. Chr3.993, KY. Chr4.460, KY. Chr8.629), potassium channel (KY. Chr2.1147), water channel (KY. Chr3.1346), tryptophan metabolism (KY. Chr11.1260), organic cation/anion/zwitterion (KY. Chr1.2519)

## Discussion

Understanding miRNA abundance dynamics and regulatory mechanisms is crucial for determining how miRNA-regulated gene expression networks contribute to phenotypic plasticity under environmental stresses. The present study investigated the co-expression of intragenic miRNAs and their host genes, as well as the regulatory functions of DEmiRNAs under recurring salinity stress in the invasive ascidian *C. robusta*. Despite the genomic nestedness of intragenic miRNAs, only 44 of 490 (9%) miRNA–host gene pairs showed co-expression (Fig. 3). Co-expression patterns were dynamically influenced by the stress course, revealing distinct miRNA–host gene pairs co-expressed at different stages of stress (Fig. 3; Supplementary Table S1). Moreover, stress time affected the expression of co-expressed DEmiRNAs (Fig. 3). Functionally, DEmiRNAs regulated biological processes, such as free amino acid metabolism, water channels, and ion transport, with time- and stage-dependent variations to maintain osmotic homeostasis. Different targets were modulated by the same or different DEmiRNAs to regulate the same osmolytes (Fig. 4; Table 1). These findings show the dynamic regulation of miRNA–host gene co-expression during recurring stresses and demonstrate that miRNAs play a critical role in regulating gene expression for osmotic balance across different stress stages. This study provides new insights into the role of miRNAs in regulating gene expression for phenotypic plasticity in response to environmental challenges during biological invasions.

### Dynamic co-expression changes during the recurring challenge

To date, studies on the co-expression between intragenic miRNAs and their host genes have primarily focused on human diseases, particularly cancer (Gao et al. 2012; Liu et al. 2019a; Zeidler et al. 2020). Although accumulating evidence has shown multiple exceptions to the co-expression pattern, most intragenic miRNA–host gene pairs have exhibited co-expression across different environments (Liu et al. 2019a, 2021). Similar co-expression among neighboring genes has been observed in several species, including *C. robusta* used in this study, emphasizing the importance of neighboring genes and their genomic architectures in determining co-expression (Chen et al. 2022). However, despite using intronic, exonic, and junction miRNAs, which are largely believed to be co-expressed with their host genes, only a small proportion of co-expressed pairs (9%, Fig. 3) were detected, significantly lower than what has been observed in humans (Liu et al. 2021) and other neighboring genes in this species (Chen et al. 2022). This finding, as well as other patterns identified in this study, illustrates that the co-expression between intragenic miRNAs and host genes is more complex than previously understood, thereby directing future investigations into the factors underlying the diverse regulations of miRNAs in ecological and evolutionary studies.

Notably, the recurrent osmotic stress did not affect the overall co-expression between all intragenic miRNAs and their hosts (Fig. 2C). Although there are no comparable cases for the co-expression between miRNAs and hosts in ecological and evolutionary studies, high salinity stresses have been shown to significantly disrupt the co-expression of neighboring genes at the whole-genome level in *C. robusta* (Chen et al. 2022). However, when examining individual miRNA–host gene pairs, dynamic changes in co-expression were observed (Fig. 3). These patterns indicate that environmental stress did not uniformly influence miRNA biogenesis through common processes affecting all miRNAs. Given that > 90% of miRNA–host pairs were expressed independently (Fig. 3), if common regulatory processes, such as cleavage, nuclear export, and associated proteins (e.g., Drosha, DGCR8, and Dicer), were involved or disrupted by stress, we expect more uniform changes in overall miRNA expression, further leading to significant changes in the overall co-expression pattern. Similar to the organization of miRNA–host gene pairs, the highly compacted *C. robusta* genome has approximately 20% of its genes arranged in operons (Chen et al. 2022; Satou et al. 2008). Although genes within an operon are co-transcribed into a single poly-cistronic pre-mRNA, they are not always co-expressed, particularly under environmental stress (Chen et al. 2022). Additional post-transcriptional regulation, particularly function-specific regulation during environmental stresses, may be the main factor driving the disrupted co-expression (Chen et al. 2022). Collectively, these function-specific regulations, rather than the general regulatory mechanisms involved in miRNA biogenesis, are potentially the primary drivers of the stress time-dependent co-expression patterns observed across recurrent salinity stresses.

To date, the detailed mechanisms underlying decoupled co-expression remain largely unknown. However, available evidence indicates several possible pathways. First, miRNAs may have their own promoters and be transcribed independently (Veronese et al. 2011). Approximately one-third of intronic miRNAs have independent promoters; these miRNAs can be transcribed either independently or dependently with their hosts (Monteys et al. 2010; Veronese et al. 2011). Second, exon skipping could account for the independent transcription observed in some cases. In the case of *miR-499* embedded in the *MYH7b* gene in humans, exon skipping introduces a premature termination codon, downregulating *MYH7b* expression, while the transcription of *miR-499* remains unaffected (Bell et al. 2010). Third, post-transcriptional regulation may contribute to the lack of co-expression. For example, pre-miR-450b is co-transcribed with its host gene *MIR503HG* in humans; however, the expressions of *miR-450b-3p* and *MIR503HG* are uncorrelated (Liu et al. 2021). Further validation of these mechanisms poses significant challenges, even in well-established systems for human disease analysis systems. These challenges include technical issues, such as accurately identifying the independent promoters of miRNAs, and the inherently complex dynamics of miRNA expression, particularly those involved in post-transcriptional regulation (Liu et al. 2019a). Further in-depth investigations should focus on validating the potential mechanisms underlying the decoupling of co-expression. RNA ligase-mediated RACE could be employed to identify the 5' TSSs and 3' ends of pri-miRNAs, confirming whether miRNAs and their host genes possess independent transcript units (Ramalingam et al. 2014). Based on diverse cases in miRNA-based studies of human diseases, the regulatory mechanisms underlying miRNA abundance are more complex and variable than previously understood. Leveraging the detailed insights from these studies, ecological and evolutionary research can further investigate the causes and consequences of miRNA-mediated functional regulations.

### Stress time-dependent functional regulations by co-expressed DEmiRNAs

Notably, osmoregulation is crucial for aquatic animals as it enables the maintenance of homeostasis across biological processes despite fluctuations in external salinity (Pourmozaffar et al. 2020; Yancey 2005). In contrast to marine osmoregulators, which typically have specialized organs, such as gills and kidneys, to maintain internal osmotic concentrations of 400 milliosmoles or lower, osmo-conformers such as *C. robusta* utilize an array of osmolytes that can be up- or downregulated to prevent osmotic shrinkage or swelling in response to external salinity changes (Sokolov et al. 2019; Yancey 2005). These osmolytes include inorganic ions, such as Na^+^, K^+^, and Ca^2+^, as well as a diverse array of organic solutes, including carbohydrates, amino acids and derivatives, methylamines and derivatives, and urea (Pourmozaffar et al. 2020; Yancey 2005). These diverse osmolytes provide osmo-conformers with multiple pathways to maintain osmotic homeostasis. However, studies have shown that some osmolytes are used broadly across various taxa, and others are restricted to specific taxonomic groups (Yancey 2005). In response to the salinity stress observed in this study, DEmiRNAs primarily regulated biological functions associated with three pathways: water transport, ion transport, and the biogenesis and metabolism of FAAs (Fig. 4). These pathways have been shown to be regulated in invasive ascidians through other mechanisms, including alternative splicing and alternative polyadenylation (Huang et al. 2023a). By integrating diverse omics data, such as miRNAome and DNA methylation profiles, functional overlaps and complementarities in response to repeated salinity stresses in *C. robusta* were observed. Additional pathways, including lipid metabolism and taurine transport, were found to be regulated by DNA methylation. Moreover, miRNAs and DNA methylation processes were involved in regulating potassium channel-related genes to mitigate salinity fluctuations; however, they targeted distinct functional genes: miRNAs regulated *KCNQ*, whereas DNA methylation modulated *KCNN* (Fu et al. 2021a). In related species such as *C. savignyi*, FAAs are preferential osmolytes for maintaining osmotic homeostasis under salinity stress (Huang et al. 2023a). Unlike the FAA metabolic processes observed in the current study, *C. savignyi* recruits the *SLC6* gene to facilitate the transport of FAAs (Huang et al. 2023a). The “all roads lead to Rome” model offers osmo-conformers increased flexibility through systemic integration of diverse regulatory mechanisms to manage environmental challenges.

In addition to the diverse osmolytes, multiple regulatory targets were involved in the same pathways, such as distinct targets for FAA metabolism and ion transport (Table 1). Both miR-9880-3p and miR-124-2-5p, which are involved in the tryptophan metabolism pathway, targeted two different genes, whereas miR-4046-5p and miR-124-2-5p regulated distinct targets within glutathione metabolism (Fig. 4). Several studies have observed a similar pattern. During two rounds of temperature stress in rice, DEmiRNAs regulated distinct targets to achieve the same function; miR-167e responded to the first stress by targeting *HSFA2*, whereas miR-1846d-5p responded to the second stress by regulating *HSF29* (Kushawaha et al. 2021). These findings reveal the presence of multiple regulatory targets within the same pathways in both animals and plants. Given that the causes and consequences of the common presence of multiple regulatory targets are largely unknown, many issues deserve further investigations, such as how multiple targets are utilized and regulated or switched during environmental challenges, whether the presence of multiple regulatory targets enhances a species’ capacity to cope with such challenges, how multiple regulatory targets evolve in conjunction with their regulators such as miRNAs, and whether variations induced by environmental stresses within and among populations contribute to selective adaptation, further advancing evolutionary processes.

The use of functional pathways regulated by DEmiRNAs exhibited a pattern independent of stress time, indicating a functional switch in osmolyte metabolism during recurrent salinity stress (Fig. 4; Table 1). Despite sharing a common strategy (e.g., ion transport) across three stress stages, the specific pathways varied: sodium channels at the S1 stage, chloride channels at the R stage, and cation channels at the S2 stage (Fig. 4). Moreover, despite the lack of relevant studies and detailed investigations into the associated mechanisms, such a switchover may be driven by the concentration limits of osmolytes. In addition to their osmotic effects, osmolytes have unique physiological roles; for example, taurine functions as an antioxidant (Jong et al. 2021). Excessive up- or downregulation of these functionally crucial solutes can disrupt homeostasis and potentially lead to detrimental effects. The switchover of osmolytes throughout the stress course may be a rebalancing among osmolytes for osmotic maintenance without affecting associated physiological processes.

## Conclusion, challenges, and future perspectives

The observed patterns of miRNA-regulated gene expression in response to environmental challenges highlight the dynamic and intricate regulatory networks underlying invasion success. During recurrent salinity stress, miRNAs play key roles in regulating vital processes that maintain osmotic homeostasis, including FAA metabolism, water channel function, and ion transport. These processes were regulated in a stress time- and stage-specific manner, demonstrating the flexible and dynamic role of miRNAs in enabling rapid physiological adjustments. The findings indicate that miRNA-mediated gene expression is crucial for phenotypic plasticity, which is critical for invasion success under rapidly changing environmental conditions.

We found a pattern of stress time- and stage-dependent decoupling and recoupling of co-expression between intragenic miRNAs and their host genes. Notably, these co-expression patterns induced a significant influence on the functional regulation of osmotic homeostasis. These findings provide valuable insights for further investigation into the mechanisms underlying the decoupling and recoupling of co-expression. However, in the current study, several technical challenges impede further investigation. These technical issues include the inherent regulatory complexity associated with miRNAs and their host genes, such as feedback loops and cross-regulation, difficulties in accurately determining the start sites for mature miRNAs, as pri-miRNA forms can extend up to 100 kb, limited genomic resources for comprehensively dissecting regulatory networks governing miRNA-mRNA interactions, and the limitation of miRNAome and transcriptome analyses in predicting miRNA biogenesis and decay. Despite these challenges, our findings initiate further exploration of the causes and consequences of miRNA-regulated phenotypic plasticity in response to environmental challenges during invasions using multiple omics datasets and developing bioinformatic tools.

## Supplementary Information

Below is the link to the electronic supplementary material.

Supplementary file1 (DOCX 21 KB)

## Data Availability

All miRNAomic and transcriptomic data have been deposited in the National Centre for Biotechnology Information (NCBI) Sequence Read Archive (SRA) database under the accession number PRJNA775866. Any additional information required to reanalyze the data reported in this paper is available from the lead contact upon request.
