# Supplementary material for: Dynamic miRNA–host gene co-expression and functional regulation in response to salinity fluctuations during biological invasions
Source: Mar Life Sci Technol. 2026 Mar 5;8(2):496–506. doi: 10.1007/s42995-026-00361-w (PMC13198604; doi:10.1007/s42995-026-00361-w)
Supplement: Supplementary file 1 — Supplementary file1 (DOCX 21 KB) [file 42995_2026_361_MOESM1_ESM.docx]

**Supplementary Table S1** Co-expressed intragenic miRNAs and corresponding host genes

| Host pair | miRNA | host gene id | host gene description |
| --- | --- | --- | --- |
| C01 | cin-miR-5596b-3p | KY. Chr1.1656 | FBLN1 (fibulin 1) |
|  |  |  |  |
| C02 | cin-miR-7385c-3p | KY. Chr14.15 | SSH2 (slingshot protein phosphatase 2) |
|  |  |  |  |
| C03 | cin-miR-1775a-3p | KY. Chr11.1321 | SMARCC2 (SWI/SNF related, matrix associated, actin dependent regulator of chromatin subfamily c member 2) |
|  |  |  |  |
| C04 | cin-miR-5153b | KY. Chr3.790 | CENPE (centromere protein E) |
|  |  |  |  |
| C05 | cin-miR-5611-5p | KY. Chr6.697 | DLG4 (discs large MAGUK scaffold protein 4) |
|  |  |  |  |
| C06 | cin-miR-63i-3p | KY. Chr9.1057 | UMOD (uromodulin) |
|  |  |  |  |
| C07 | cin-miR-7883b-3p | KY. UAContig3.156 | CNOT8 (CCR4-NOT transcription complex subunit 8) |
|  |  |  |  |
| C08 | cin-miR-317 | KY. Chr9.306 | PARP14 (poly (ADP-ribose) polymerase family member 14) |
|  |  |  |  |
| C09 | cin-miR-10021a-5p | KY. Chr3.783 | SLC31A1 (solute carrier family 31 member 1) |
|  |  |  |  |
| C10 | cin-miR-306 | KY. Chr4.257 | AKT1 (AKT serine/threonine kinase 1) |
|  |  |  |  |
| C11 | cin-miR-2064a | KY. Chr1.1383 | DDX58 (DExD/H-box helicase 58) |
|  |  |  |  |
| C12 | cin-miR-4098-5p | KY. Chr8.1347 | MCMBP (minichromosome maintenance complex binding protein) |
|  |  |  |  |
| C13 | cin-miR-2692b | KY. Chr9.375 | ID2 (inhibitor of DNA binding 2) |
|  |  |  |  |
| C14 | cin-miR-4046-5p | KY. Chr13.448 | CCDC18 (coiled-coil domain containing 18) |
|  |  |  |  |
| C15 | cin-miR-9880-3p | KY. Chr2.42 | DDX5 (DEAD-box helicase 5) |
|  |  |  |  |
| C16 | cin-miR-7370a-5p | KY. Chr4.1292 | NF1 (neurofibromin 1) |
|  |  |  |  |
| C17 | cin-miR-7713-5p | KY. Chr1.1136 | NFE2L1 (nuclear factor, erythroid 2 like 1) |
|  |  |  |  |
| C18 | cin-miR-306-5p | KY. Chr3.646 | SUPT7L (SPT7 like, STAGA complex gamma subunit) |
|  |  |  |  |
| C19 | cin-miR-8362-3p | KY. Chr3.292 | SHBG (sex hormone binding globulin) |
|  |  |  |  |
| C20 | cin-miR-8205b-3p | KY. Chr3.781 | TMEM206 (transmembrane protein 206) |
|  |  |  |  |
| C21 | cin-miR-4220-5p | KY. Chr11.324 | PIWIL1 (piwi like RNA-mediated gene silencing 1) |
|  |  |  |  |
| C22 | cin-miR-4017-5p | KY. Chr1.1420 | RPL8 (ribosomal protein L8) |
|  |  |  |  |
| C23 | cin-miR-1497-5p | KY. Chr9.1057 | UMOD (uromodulin) |
|  |  |  |  |
| C24 | cin-miR-4081-5p | KY. Chr2.1565 | TECTA (tectorin alpha) |
|  |  |  |  |
| C25 | cin-miR-7868-5p | KY. Chr6.677 | PIGO (phosphatidylinositol glycan anchor biosynthesis class O) |
|  |  |  |  |
| C26 | cin-miR-4811a-5p | KY. Chr4.472 | CIC (capicua transcriptional repressor) |
|  |  |  |  |
| C27 | cin-miR-5596f-3p | KY. Chr1.1542 | TP53I3 (tumor protein p53 inducible protein 3) |
|  |  |  |  |
| C28 | cin-miR-4045-5p | KY. Chr1.724 | EBF3 (EBF transcription factor 3) |
|  |  |  |  |
| C29 | cin-miR-828-3p | KY. Chr14.34 | NOD1 (nucleotide binding oligomerization domain containing 1) |
|  |  |  |  |
| C30 | cin-miR-582b-3p | KY. Chr12.480 | KAT6A (lysine acetyltransferase 6A) |
|  |  |  |  |
| C31 | cin-miR-4055-5p | KY. Chr7.362 | KLHL30 (kelch like family member 30) |
|  |  |  |  |
| C32 | cin-miR-5349b-5p | KY. Chr7.101 | FIGN (fidgetin, microtubule severing factor) |
|  |  |  |  |
| C33 | cin-miR-5813 | KY. Chr5.104 | GLUD2 (glutamate dehydrogenase 2) |
|  |  |  |  |
| C34 | cin-miR-4002-5p | KY. Chr7.879 | POLR1A (RNA polymerase I subunit A) |
|  |  |  |  |
| C35 | cin-miR-4144-5p | KY. Chr10.596 | ZNF839 (zinc finger protein 839) |
|  |  |  |  |
| C36 | cin-miR-4030-5p | KY. Chr3.1131 | SRCAP (Snf2 related CREBBP activator protein) |
|  |  |  |  |
| C37 | cin-miR-124-2-5p | KY. Chr7.1017 | OSBP2 (oxysterol binding protein 2) |
|  |  |  |  |
| C38 | cin-miR-4729 | KY. UAContig9.44 | CARF (calcium responsive transcription factor) |
|  |  |  |  |
| C39 | cin-miR-7669-3p | KY. Chr3.102 | MS4A12 (membrane spanning 4-domains A12) |
|  |  |  |  |
| C40 | cin-miR-4073-5p | KY. Chr13.448 | CCDC18 (coiled-coil domain containing 18) |
|  |  |  |  |
| C41 | cin-miR-789b | KY. Chr4.257 | AKT1 (AKT serine/threonine kinase 1) |
|  |  |  |  |
| C42 | cin-miR-4093-5p | KY. Chr1.907 | OSBPL9 (oxysterol binding protein like 9) |
|  |  |  |  |
| C43 | cin-miR-182-3p | KY. Chr7.849 | EPHB3 (EPH receptor B3) |
|  |  |  |  |
| C44 | cin-miR-5678 | KY. Chr10.253 | MRPL54 (mitochondrial ribosomal protein L54) |
